# Extracellular Vesicles: Novel Roles in Neurological Disorders

**DOI:** 10.1155/2021/6640836

**Published:** 2021-02-17

**Authors:** Qian Jin, Peipei Wu, Xinru Zhou, Hui Qian, Wenrong Xu

**Affiliations:** ^1^Key Laboratory of Laboratory Medicine of Jiangsu Province, School of Medicine, Jiangsu University, 212013 Zhenjiang, Jiangsu, China; ^2^Zhenjiang Key Laboratory of High Technology Research on Exosomes Foundation and Transformation Application, Jiangsu Key Laboratory of Medical Science and Laboratory Medicine, School of Medicine, Jiangsu University, 301 Xuefu Road, Zhenjiang, Jiangsu 212013, China; ^3^Department of Laboratory Diagnostics, Changhai Hospital, Second Military Medical University, Shanghai 200433, China

## Abstract

Exosomes are small extracellular vesicles (EVs) secreted by almost all cells, which have been recognized as a novel platform for intercellular communication in the central nervous system (CNS). Exosomes are capable of transferring proteins, nucleic acids, lipids, and metabolites between neurons and glial cells, contributing to CNS development and maintenance of homeostasis. Evidence shows that exosomes originating from CNS cells act as suppressors or promoters in the initiation and progression of neurological disorders. Moreover, these exosomes have been shown to transfer molecules associated with diseases through the blood-brain barrier (BBB) and thus can be detected in blood. This unique feature enables exosomes to act as potential diagnostic biomarkers for neurological disorders. In addition, a substantial number of researches have indicated that exosomes derived from mesenchymal stem cells (MSCs) have repair effects on neurological disorders. Herein, we briefly introduce the roles of exosomes under physiological and pathological conditions. In particular, novel roles of exosomes as potential diagnostic biomarkers and therapeutic tools for neurological disorders are highlighted.

## 1. Introduction

Neurological disorders are a series of debilitating diseases that affect the nervous system, which mainly include acute central nervous system (CNS) injury such as spinal cord injury (SCI), traumatic brain injury (TBI), and stroke, as well as neurodegenerative diseases like Alzheimer's disease (AD) and Parkinson's disease (PD) [1, 2]. Currently, the diagnosis of acute CNS injury largely depends on clinical manifestations and imageological examinations, including computed tomography (CT) imaging, magnetic resonance imaging (MRI), and X-ray [3]. With regard to acute CNS injury, surgery remains a clinical emergency measure, though there is no reparative strategy to cure it [4]. At the same time, cerebrospinal fluid (CSF) puncture and positron emission tomography (PET) imaging are recommended as diagnostic tools for neurodegenerative diseases [5]. However, excessive economic costs limit the clinical utilization rate of PET, while CSF puncture may induce surgical trauma [6]. Besides, as neurodegenerative diseases lack specific symptoms, most patients are diagnosed in the middle or late stages, and treatment can only delay the progression of the diseases [7]. Therefore, it is an urgency to explore specific and effective approaches for the diagnosis and treatment of neurodegenerative diseases.

Extracellular vesicles (EVs) are subcellular components produced by the paracrine pathway of cells, mainly including apoptotic bodies (500-2000 nm in diameter), microvesicles (200-2000 nm in diameter), and exosomes (30-200 nm in diameter) [8]. Exosomes are defined as small lipid bilayer-enclosed EVs [9], which play a vital role in the communication between neurons and glial cells [10]. In addition, CNS cell-derived exosomes can transfer disease-related substances through the blood-brain barrier (BBB) and thus can be detected in patients' blood [11]. This characteristic makes exosomes to become potential valuable diagnostic biomarkers for neurological disorders. Importantly, a large number of studies have confirmed that exosomes derived from mesenchymal stem cells (MSCs) possessed obvious repair effects on neurological disorders in many animal models [12]. Therefore, exosomes are expected to become candidate clinical therapeutic tools.

In this review, we summarize the roles of exosomes as mediators of intercellular communication under physiological and pathological conditions. We also present recent literatures on the potential application of exosomes in the diagnosis of neurodegenerative diseases, and finally, the current knowledge around the prospects of MSC-derived exosomes as therapeutic tools of neurological disorders are discussed.

## 2. Biosynthesis, Secretion, Uptake, and Components of Exosomes

**Figure 1:**
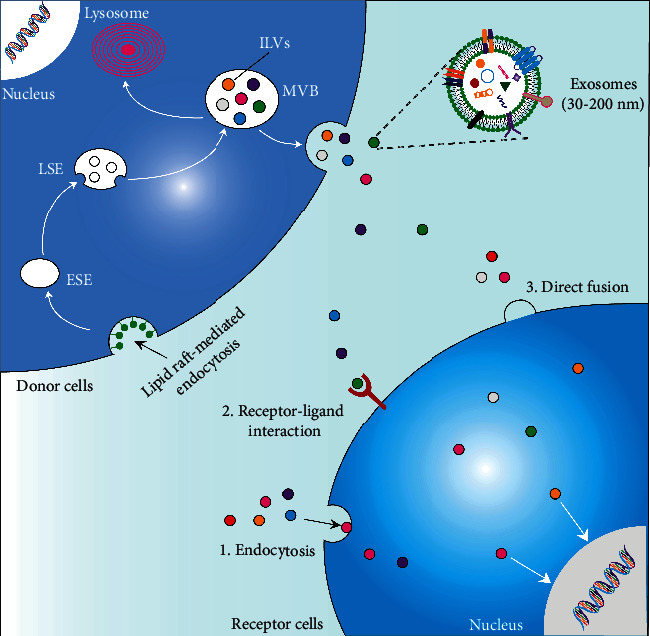
Biosynthesis, secretion, uptake, and components of exosomes. Exosome biosynthesis begins in the endosomal pathway. The cytoplasmic membrane invaginates to form early sorting endosomes (ESEs) that could mature into late sorting endosomes (LSEs), and the membrane of LSEs invaginates to form multivesicular bodies (MVBs) that contain several intraluminal vesicles (ILVs). MVBs can fuse with the plasma membrane to secret ILVs as exosomes or fuse with lysosomes to be degraded. Exosomes transfer specific proteins, nucleic acids, lipids, and metabolites to receptor cells by endocytosis, receptor-ligand interaction, and direct membrane fusion.

Exosome biosynthesis is a tightly controlled process including three stages: endocytosis, multivesicular bodies (MVBs) formation, and exosome secretion [13] (Figure 1). Generated through plasma membrane endocytosis, early sorting endosomes (ESEs) mature into late sorting endosomes (LSEs). Subsequently, the invagination of the LSE membrane can form MVBs that contain several intraluminal vesicles (ILVs) [14]. MVB biosynthesis is either connected to the physical characteristics and lipid component of endosome raft microdomains, or related to the goods sorting probably through the endosomal sorting complex required for transport- (ESCRT- ) dependent and -independent signals [15, 16].

When getting matured, MVBs can fuse with the plasma membrane to release exosomes or fuse with lysosomes to get degraded [17]. Multiple molecular motor kinesins drive MVBs to target the plasma membrane. The critical step of exosome secretion is to dock MVBs with SNARE outside the membrane [18]. These processes are modulated by RAB family proteins (RAB7, RAB11, RAB27, and RAB35) [19] and RAL-1 in the family of Ral guanosine triphosphatase (GTPase) [20]. The precise processes and regulatory mechanisms of exosomal biosynthesis and secretion are still in need of further exploration. Exosomes are released to extracellular space, which can influence cells in the extracellular matrix. The exosomes mainly transmit information and cargoes to recipient cells through three ways: endocytosis, direct fusion with the plasma membrane, and receptor-ligand interaction [17]. Exosomes mainly contain proteins, lipids, nucleic acids, and metabolites. Different components of exosomes may reflect their cellular origin, as well as the physiological and pathological state of the microenvironment [21].

## 3. Isolation, Detection, and Identification of Exosomes

Exosomes can be isolated from different body fluids and cells. According to the physical and chemical characteristics of exosomes, different extraction methods are established, and researchers can choose appropriate separation methods according to purity requirement. Common isolation methods are ultracentrifugation [22], size exclusion chromatography [23], immune-affinity capture [24], ultrafiltration [25], commercial kit [26], and microfluidics [27]. Each method has its own advantages, disadvantages, and application scopes (Table 1), among which the ultracentrifugation method is regarded as the gold standard of exosome extraction and widely used by researchers [28]. However, the method requires expensive instruments and time-consuming operation, and the integrity and biological activity of exosomes cannot be guaranteed; so, it cannot be applied to clinical detection [29]. Currently, there is no method that can obtain exosomes with high yield, high purity, and good integrity to meet various applications; so, researchers need to choose different extraction methods or method combinations according to their own experimental requirements.

The extracted exosomes can be detected and identified in accordance with their biochemical properties. The morphology and particle size of exosomes are detected via scanning electron microscopy [30], transmission electron microscopy [31], and atomic force microscopy [32]. Dynamic light scattering, nanoparticle tracking analysis, and tunable resistive pulse sensing are frequently applied for rapid determination of the exosome particle size and concentration [33]. Exosomes perform biological functions mainly depending on their contents. Traditional technologies for exosome protein detection mainly include Western blotting (WB) and enzyme-linked immunosorbent assays (ELISA), and high-throughput sequencing and PCR amplification are used for exosome RNA detection [8]. In addition, emerging technologies such as microfluidic chips [34], droplet digital PCR [35], and ion-exchange nanomembrane detection [36] are also used to characterize exosomes.

## 4. Roles of Exosomes as Intercellular Communication Mediators under Physiological and Pathological Conditions

**Figure 2:**
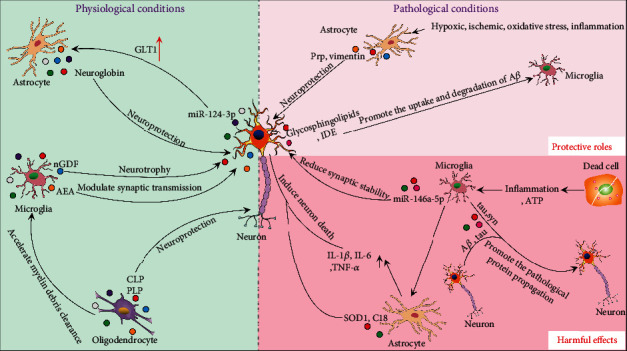
Roles of exosomes as intercellular communication mediators under physiological and pathological conditions. Under physiological or pathological conditions, cells can multidirectionally deliver substances and exchange information through exosomes. nGDF: nervous growth/differentiation factor; AEA: *N*-arachidonoylethanolamine; PLP: proteolipid protein; CNP: 2′3′-cyclic-nucleotide-phosphodiesteras; PrP: prion protein; A*β*: amyloid *β*; IDE: insulin-degrading enzyme; SOD1: mutant superoxide dismutase.

Exosomes, which are secreted by neurons, astrocytes, microglia, and oligodendrocytes, mediate intercellular communication (Figure 2). Under physiological conditions, communication between neurons and glial cells contributes to CNS development and maintenance of homeostasis [37]. Through carrying signaling information, neuronal exosomes can regulate the development of neural circuit [38]. Moreover, neuronal exosomes can transfer neuron-specific cargoes to glial cells and regulate their functions as well. For instance, neuronal exosomes can carry miR-124-3p to astrocytes and further upregulate the glutamate transporter GLT1 in astrocytes [39]. Other studies indicate that exosomes derived from astrocytes can transfer neuroglobin to neurons, which protects neurons against cell insult [40]. Exosomes from microglia can transfer nervous growth/differentiation factor (nGDF) to neurons, which reveals obvious neurotrophic activities [41], and they can also carry *N*-arachidonoylethanolamine (AEA) by targeting GABAergic neurons to modulate synaptic transmission [42]. Microglia can take up oligodendrocytes-derived exosomes to clear myelin debris [43]. Moreover, exosomes from oligodendrocytes contain major myelin proteolipid protein (PLP) and 2′3′-cyclic-nucleotide-phosphodiesterase (CNP), can also support neurons, and maintain the balance of myelin proteins and lipids [44].

Neurological disorders may cause ischemia, hypoxia, oxidative stress, and inflammation, which can even lead to neurons death. Under pathological conditions, exosomes may be angel or demon [45]. On one hand, exosomes play protective roles in protecting neurons or clearing up pathological proteins. For instance, astrocyte-derived exosomes transfer prion protein (PrP) to neurons, which improves the survival of neurons under hypoxic and ischemic conditions [46]. When SCI occurs, astrocytes release vimentin via exosomes to protect neurons [47]. Moreover. AD is characterized by the deposition of amyloid *β* (A*β*) and phosphorylated tau protein. Neuronal exosomes carry abundant glycosphingolipids to capture extracellular A*β* and drive microglia to uptake and degrade A*β* [48, 49]. Furthermore, insulin-degrading enzyme (IDE) enriched in exosomes can also drive microglia to degrade A*β* [50].

On the other hand, exosomes can accelerate the progression of diseases through the propagation of inflammation and pathological proteins within CNS. Neurological disorders could cause inflammation, during which microglia as the main immune cells are firstly activated. However, proinflammatory microglia are insufficient to kill neurons; so, they transfer exosomes to astrocytes, which induce the activation of A1 astrocytes, leading to the damage of neurons [51, 52]. In addition, dead cells release ATP and drive microglia to the injury sites. ATP stimulation changes the proteome of exosomes derived from microglia, obviously causing the activation of astrocytes and the upregulation of IL-1*β*, IL-6, and TNF-*α* [53]. What is more, astrocyte-derived exosomes could transfer mutant superoxide dismutase (SOD1), proapoptotic C18 ceramide, and complement proteins to neurons and lead to the damage of neurons [54–56]. Studies have also demonstrated that exosomes can transfer toxic A*β* and tau between neurons, resulting in neuron death and pathological protein propagation [57, 58]. Meanwhile, microglia can also spread tau by secreting exosomes [59]. PD is the second most common neurodegenerative disease in the pathogenesis of which DJ-1 and *α*-synuclein (*α*-syn) are involved. Exosomes may provide an ideal environment for the *α*-syn aggregation [60], and microglial exosomes facilitate the *α*-syn transmission [61]. Moreover, exosomes enriched with *α*-syn can promote the death of recipient neurons [62]. Additionally, miRNAs are also packaged in exosomes and are involved in the pathogenesis of neurological disorders. For instance, miR-15 may suppress the expression of beta-secretase 1 (BACE1) and abnormal phosphorylation of tau, but the level of miR-15 in AD patients' circulating exosomes is low, which reflects the correlation between exosomal miRNAs and progression of AD [63]. In addition, miR-137 is upregulated in PD mice's serum exosomes and can induce neuronal oxidative stress [64]. Other studies show that microglia deliver miR-146a-5p to neurons via exosomes, which reduces the dendritic spine density and synaptic stability by inhibiting the expression of presynaptic synaptotagmin1 (Syt1) and postsynaptic neuroligin1 (Nlg1) in neurons [65]. In summary, cells can multidirectionally deliver substances and exchange information through exosomes, accelerating or delaying the progression of neurological disorders.

An understanding of the roles of exosomes in CNS cell communication will be conductive to finding the targets for the diagnosis and treatment of neurological disorders. A question why exosomes play different roles under pathological conditions is crucial to be explored. The possible factor is the different components of exosomes [45]. Therefore, further researches may aim at identifying the detrimental and beneficial components of exosomes, so that researchers can artificially load disease-suppressing cargoes into exosomes to alleviate neurological disorders. Moreover, CNS cell-derived exosomes can shuttle specific cargoes through the BBB and, as a result, can be detected in blood. The cargoes of exosomes indicate the pathological conditions for the source of cells and are tightly associated with the stages of neurological disorders. Substantial researches have been devoted to exploring the translational potential of exosomes as reliable diagnostic biomarkers for neurodegenerative diseases.

## 5. Potential Diagnostic Biomarkers of Exosomes in Neurodegenerative Diseases

CSF makes contact with the brain and spinal cord, which allows the former to reflect the CNS pathophysiological changes [66]. However, as a reliable specimen, CSF is not easily available, and lumbar puncture can induce surgical trauma. Consequently, compared with CSF, peripheral blood may be an ideal source for the diagnosis of neurodegenerative diseases [67].

**Figure 3:**
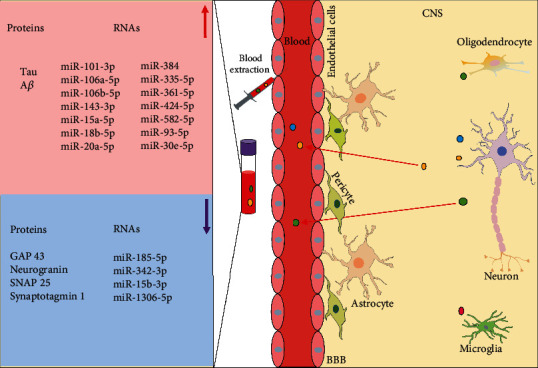
Exosomes as diagnostic biomarkers in the AD patients' peripheral blood. There are differentially expressed proteins and RNAs in the exosomes of the AD patients' blood. CNS: central nervous system; BBB: blood-brain barrier; A*β*: amyloid *β*; GAP43: growth-associated protein 43; SNAP25: synaptosome-associated protein 25.

BBB, formed by tight junction of brain endothelial cells and coverage of pericytes, astrocyte end feet, and capillary basement membrane, is a selective biological barrier between the blood and neuronal tissue. It prevents brain-derived toxic substances from entering into peripheral blood and blood-derived pathogens into CNS, but selectively transport essential metabolites such as water, glucose, and amino acids into CNS to maintain the tissue function [68]. With their unique member lipid and protein composition, exosomes can easily penetrate through BBB [69]. Recent studies have reported that CNS cell-derived exosomes could transfer substances associated with neurodegenerative diseases through BBB so that it could be detected in patients' blood (Figure 3). Zhao et al. have demonstrated that the levels of DJ-1 and *α*-syn from neural-derived exosomes are significantly higher in PD patients' plasma than those of healthy people [70]. Furthermore, it has been shown that the levels of A*β* and tau in neuronal-derived exosomes are higher in AD patients' blood and greatly correlated with those in CSF [5, 71]. Commercial kit and immunoprecipitation methods have been used to isolate the neuronal-derived exosomes in the blood. Neural cell adhesion molecule (NCAM) antibody and L1 cell adhesion molecule (L1CAM) antibody can be adopted to enrich neuronal-derived exosomes. Above studies suggest that neuronal-derived exosomes may be used as potential diagnosis biomarkers.

Neurodegenerative diseases are chronic progressive diseases, the pathological changes of which are permanent. In many cases, by the time when patients have symptoms and visit hospitals, diseases probably have already progressed to the middle or late stages, when treatment can only slightly function to postpone the development of diseases but cannot fundamentally cure the damage to the nervous system [72]. Hence, to search for early diagnostic biomarkers of neurodegenerative diseases is particularly helpful. Jia et al. found that concentrations of the growth-associated protein 43 (GAP43), neurogranin, synaptosome-associated protein 25 (SNAP25), and synaptotagmin 1 from neuronal-derived exosomes were lower in AD patients' blood, with which researchers may predict AD at the asymptomatic stage [73].

RNAs are selectively packaged into exosomes by complex mechanisms during the formation of exosomes and can be extracted from blood exosomes by commercial kit. In serum exosomes of AD patients, miRNAs (miR-15, miR-185-5p, and miR-342-3p), were downregulated, and as their predicted target, APP was abnormally elevated in the AD brains, while miRNA-mRNA interaction caused less miRNAs (miR-15, miR-185-5p, and miR-342-3p) which to be sorted into exosomes [63]. Besides, Cheng et al. have selected sixteen AD-specific miRNAs (miR-101-3p, miR-106a-5p, miR-106b-5p, miR-1306-5p, miR-143-3p, miR-15a-5p, miR-15b-3p, miR-18b-5p, miR-20a-5p, miR-30e-5p, miR-335-5p, miR-342-3p, miR-361-5p, miR-424-5p, miR-582-5p, miR-93-5p) from serum exosomes as candidate diagnostic and prognostic biomarkers by high-throughput next-generation sequencing and qRT-PCR between AD patients and healthy people. Compared with healthy controls, the expression of miR-342-3p, miR-15b-3p, and miR-1306-5p was lower in serum exosomes of AD patients, while that of other 13 miRNAs was higher [74]. As the clinical manifestations of some neurodegenerative diseases are similar, it is particularly important to find specific diagnostic biomarkers that is particularly important. On that note, it is inspiring as a research which recently demonstrated that miR-384 was highly expressed in AD patients' serum exosomes and had high specificity to rule out other diseases, such as vascular dementia (VaD) and PD with dementia (PDD) [75].

All the above reports suggest that exosomes may be potential diagnostic biomarkers in neurodegenerative diseases. In effect, the presentation of biomarkers in the blood is even earlier than clinical symptoms; so, to find higher sensitive biomarkers for screening and early diagnosis of neurodegenerative diseases has great value. Currently, exosome-based diagnostic platforms are in a promising stage of clinical transformation. Further experiments are needed to span the long standing gap between initial discovery of exosomal biomarkers and clinical application.

## 6. Therapeutic Potentials of MSC-Derived Exosomes in Neurological Disorders

Neurological disorders occasion a series of sensorimotor dysfunctions and pathological abnormalities of CNS. In the CNS of mature mammalian, injured axons cannot regenerate spontaneously because of the prohibitive microenvironment, poor nerve regeneration capacity, and a lack of growth stimulating factors [76]. Currently, surgical intervention and drug treatment can alleviate diseases but cannot cure them absolutely, making the treatment of neurological diseases still a major problem that has not yet been overcome in the medical field [77]. Therefore, it is imperative to explore novel therapeutic means for the eradication of neurological disorders.

**Figure 4:**
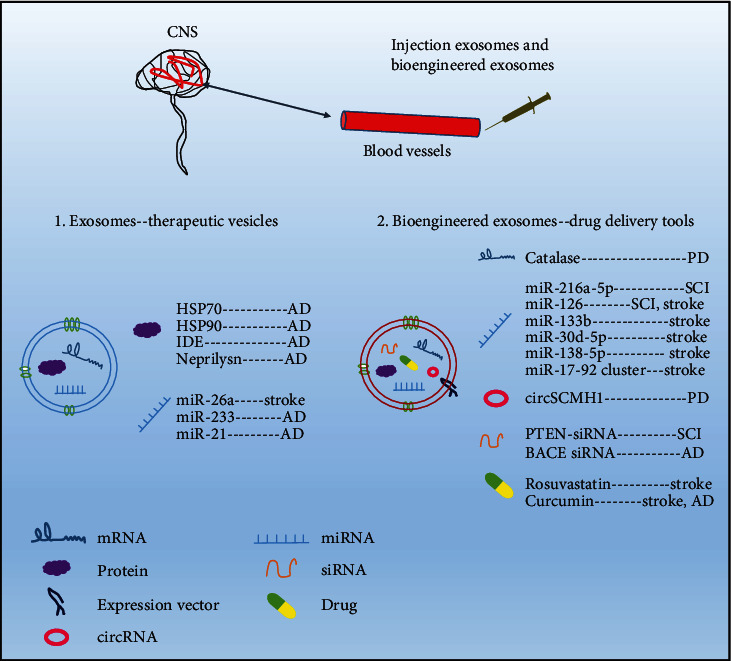
Exosomes act as natural vesicles or drug delivery tools to treat neurological disorders. Exosomes can carry their own substances or therapeutic exogenous cargoes through BBB into the damaged sites of CNS, with low toxicity and immunogenicity. CNS: central nervous system; BBB: blood-brain barrier; SCI: spinal cord injury; AD: Alzheimer's disease; PD: Parkinson's disease; IDE: insulin-degrading enzyme; HSP: heat shock protein; PTEN: phosphatase and tensin homolog; BACE1: beta-secretase 1.

From a therapeutic point of view, MSCs have been widely used in regenerative medicine [78]. MSC therapy for neurological disorders has exhibited neuroprotection potentials in laboratories and clinical studies [79]. MSCs, as adult stem cells derived from mesoderm, have self-renewal and multidirectional differentiation capacity. They are widely distributed in various tissues and organs throughout the body, such as bone marrow, fat, pulp, and umbilical cord [80]. Rather than replacing dying neurons, MSCs exert beneficial effects largely by the paracrine pathway to improve the damaged microenvironment. Evidence shows that exosomes play key roles in the paracrine pathway and exhibit neuroprotection effects [81]. Exosomes have been tapped as potential tools for the treatment of neurological disorders. On the one hand, they are natural vesicles carrying proteins, lipids, and RNAs, with the ability to cross BBB and mediate changes in gene expressions in the CNS recipient cells. On the other hand, bioengineered exosomes are manufactured by artificially loading therapeutic cargoes (such as therapeutic siRNAs [82], miRNAs [83], and drugs [84]) into exosomes, which could open a new field in drug delivery systems, with a number of advantages. Firstly, exosomes protect exogenous drugs from degradation. Next, brain-specific delivery increases the delivery efficiency of drugs. Matthew et al. firstly report the protocol of the generation of targeted exosomes by transfection of an expression vector, and the bioengineered exosomes can cross BBB and specifically delivery therapeutic siRNAs into the mice brain [85]. In addition, bioengineered exosomes have little toxicity or immunogenicity in treated animals, which mainly depend on their cellular sources. For instance, immature dendritic cells and MSC express immunological inertness, so that immature dendritic cells and MSC-derived exosomes have few immune stimulatory molecules [82]. In conclusion, exosomes play therapeutic roles mainly by delivering drugs or their nature therapeutic characteristics (Figure 4). Next, we will discuss the therapeutic potentials of MSC-derived exosomes in neurological disorders (Table 2).

### 6.1. Therapeutic Potentials of MSC-Derived Exosomes in Acute CNS Injury

**Figure 5:**
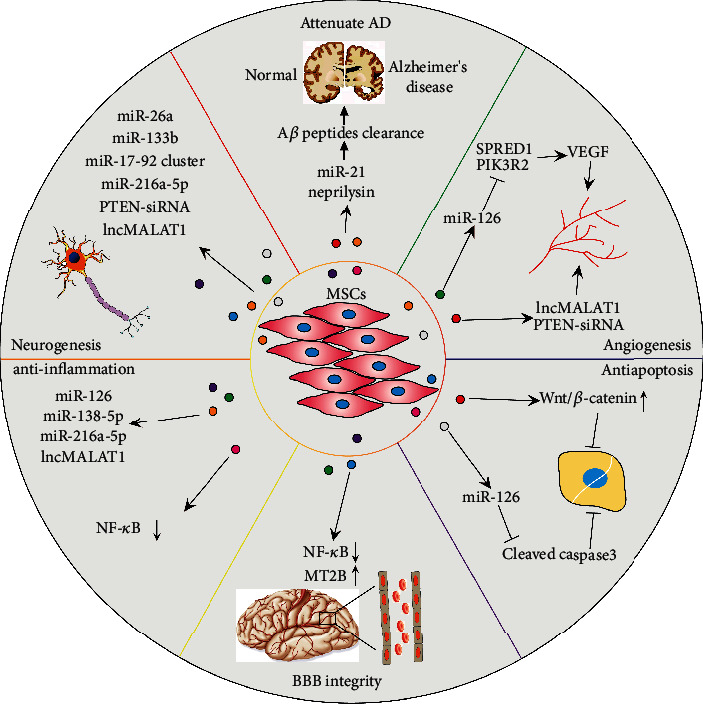
MSC-derived exosomes as potential treatment tools for neurological disorders. Exosomes facilitate the functional recovery of neurological conditions by promoting neurogenesis, angiogenesis, BBB integrity, suppressing inflammation, apoptosis, and attenuating disease progression. MSCs: mesenchymal stem cells; AD: Alzheimer's disease; A*β*: amyloid *β*; lncMALAT1: long noncoding RNA MALAT1; SPRED1: sprouty-related EVH1 domain-containing protein 1; PIK3R2: phosphoinositide-3-kinase regulatory subunit 2; PTEN-siRNA: phosphatase and tensin homolog small interfering RNA; NF-*κ*B: nuclear factor-*κ*B; BBB: blood-brain barrier; VEGF: vascular endothelial growth factor.

Acute CNS injury is probably caused by sudden external injury, tumor compression, or other factors, which leads to consecutive pathological changes of the brain and spinal cord, as well as incomplete or complete functional losses below the site of injury. Primary injury physically destroys the original structure of the brain and spinal cord, usually initiating cascaded secondary injury that enlarges the initial area of damage. In the acute phase, the death of neural cells and axon degeneration occurs at the center of lesion, where microglia are recruited to induce immune responses [86]. In the subacute stage, reactive astrocytes are activated, and inflammation is aggravating. Meanwhile, myelin debris is removed by microglia, resulting in cavity formation and then inflammation diffuses. In the chronic stage, astrocytes overgrow to form glia scars, which limits the spread of inflammation but physically blocks axon regeneration [87]. Accordingly, therapeutic approaches could aim at multiple aspects of the damage (Figure 5).

#### 6.1.1. Therapeutic Potentials of MSC-Derived Exosomes in SCI

Researchers usually apply impactors to construct incomplete SCI models and extract exosomes derived from MSCs by the ultracentrifugation method. Huang et al. have found that bone marrow MSC- (BMMSCs-) derived exosomes suppressed inflammation, attenuated apoptosis, promoted angiogenesis, and improved functional recovery in a rat model of SCI [88]. In addition, it has been verified that BMMSC-derived exosomes could inhibit neuronal apoptosis and promote the recovery of the spinal cord function by activating the Wnt/*β*-catenin signaling pathway [89] which is involved in diverse physiological processes, and its activation also contributes to neurogenesis and functional recovery after CNS injury [90].

Pericytes can participate in the composition of neurovascular units. When SCI happens, blood vessels are destroyed, and pericytes detach from neurovascular wall, resulting in the impairment of blood-spinal cord barrier (BSCB) at the lesion site. The nuclear factor-*κ*B (NF-*κ*B) signaling pathway is involved in the regulation of cellular proliferation and migration, as well as the inflammatory response. Interestingly, EVs isolated from BMMSCs could inhibit the abnormal migration of pericytes and maintain the integrity of the BSCB via suppressing the activation of the NF-*κ*B signaling pathway [91]. As a target gene of NF-*κ*B, C3 plays an important role both in the classical complement activation pathway and the alternative activation pathway. When SCI occurs, the expression of C3 was significantly upregulated in neurotoxic A1 astrocytes. Exosomes derived from BMMSCs could suppress the expression of C3 probably by inhibiting the activation of the NF-*κ*B signaling pathway [92, 93]. Liu et al. combined differential ultracentrifugation and density gradient centrifugation to extract exosome, and 30% sucrose/D2O cushion liquid were applied in the isolation process. These studies clearly indicate the roles of BMMSC-derived exosomes as therapeutic tools for SCI.

Human umbilical cord MSCs (hucMSCs) are also widely used in tissue regeneration [94]. Sun et al. revealed that exosomes derived from hucMSCs could promote spinal cord functional recovery after SCI by inhibiting inflammation. In this study, 20 *μ*g and 200 *μ*g exosomes were injected through the tail vein into mice with SCI, compared with 20 *μ*g exosomes, and 200 *μ*g exosomes had more obvious healing powers [95]. HucMSC-derived exosomes could reduce the activation of A1 astrocytes and function as anti-inflammation mediators via the regulation of the Nrf2/NF-*κ*B signaling pathway [96]. This study suggests that hucMSC-derived exosomes may be a potential therapy tool for the treatment of neurological disorders related to inflammation.

Compared with natural exosomes, bioengineered exosomes have better reparative effects, which are applied in the drug delivery field. We can manufacture bioengineered exosomes through directly modifying exosomes or modifying MSCs in an indirect way. By altering the culture conditions of MSCs or modifying MSCs with gene manipulation, a desired gene could be overexpressed in the exosomes secreted by MSCs. For instance, miR-216a-5p was enriched in exosomes derived from hypoxic preconditioned BMMSCs, which exhibited better anti-inflammation and functional recovery effects on SCI by mediating the TLR4/NF-*κ*B/PI3K/AKT signaling cascade pathways involved in the modulation of microglia polarization [97]. Some therapeutic miRNAs, such as miR-133b and miR-126, known to promote neurogenesis [98, 99], were transfected into MSCs. Exosomes produced by the modified MSCs could deliver the therapeutic miRNAs into lesion sites and promote the recovery of the neurological function. For example, exosomes from BMMSCs overexpressed with miR-133b significantly promoted neurogenesis by activating the ERK1/2, STAT3, and CREB signaling pathway; in this study, compressed SCI was inflicted by 35 g closing force for 60 s at the T10 level [100]. Exosomes derived from BMMSCs overexpressed with miR-126 suppressed the apoptosis of cells and promoted the generation of nerves and blood vessels. Further analysis indicated that miR-126 promoted the angiogenesis probably by inhibiting the expression of sprouty-related EVH1 domain-containing protein 1 (SPRED1) and phosphoinositide-3-kinase regulatory subunit 2 (PIK3R2) [101].

Moreover, exosomes could directly be modified by chemical, physical, and biological approaches. Guo et al. loaded phosphatase and tensin homolog (PTEN) small interfering RNA (PTEN-siRNA) into exosomes by passive incubation, which promoted neurogenesis, angiogenesis, and significant functional recovery of complete SCI rats [102]. In addition to passive loading, some active loading methods such as electroporation, extrusion, and liposome-mediated membrane fusion have also been recommended [8].

The delivery mode of exosomes in vivo also affects the therapeutic effects to some extent. As the most common mode, single or repeated intravenous injection during the treatment period is adopted. Recently, intranasal infusion emerges as a noninvasive, rapid, and convenient procedure. Guo et al. verified that exosomes can pass the BBB and be better retained at the site of injury by means of repeated intranasal infusion [102]. At the same time, Li et al. immobilized exosomes derived from MSCs in a peptide-modified adhesive hydrogel and transplanted the latter into the lesion site, which exhibited its abilities of efficient retention and sustained release [103]. These results collectively indicate that exosomes and bioengineered exosomes both have beneficial effects and application prospects for the treatment of SCI.

#### 6.1.2. Therapeutic Potentials of MSC-Derived Exosomes in TBI

By modulating microglia polarization, exosomes derived from BMMSCs could suppress inflammation to promote functional recovery of TBI mice [104]. Moreover, adipose mesenchymal stem cells- (AMSCs-) derived exosomes, containing long noncoding RNA MALAT1 (lncMALAT1), have significantly promoted functional recovery by boosting neurogenesis, restraining inflammation and apoptosis [105].

Zhang et al. have uncovered better reparative effects of exosomes isolated from MSCs cultured on 3-dimensional (3D) collagen scaffolds on rat models of TBI which was constructed by the controlled cortical impact method. In this study, exosomes were extracted by commercial kit [106]. Recently, engineered exosomes isolated from 3D-cultured MSCs have emerged as rising therapy tools applied in regeneration medicine [107]. The 3D scaffolds are comprised of biodegradable and biocompatible materials, which influence cell growth and functional activities [108]. In addition, various nerve growth factors can also be utilized to modify MSCs. Xu et al. have pretreated BMMSCs with brain-derived neurotrophic factor (BDNF), which made the exosomes overexpress miR-216a-5p. The engineered exosomes had obvious therapeutic effects on TBI by promoting neurogenesis and controlling inflammation [109].

#### 6.1.3. Therapeutic Potentials of MSC-Derived Exosomes in Stroke

Stroke is often manifested as hemorrhagic and ischemic ones with a high fatality rate and disability rate. Currently, exosomes as cell-free therapy tools are emerging for the treatment of stroke. In a rat cerebral hemorrhage stroke model, AMSC-derived exosomes functioned as paracrine substances to promote functional recovery, remodel neurovascular, and sprout axonal [110]. In a rat cerebral ischemic stroke model, urine MSC- (UMSCs-) derived exosomes enhanced neurogenesis partly by transferring miR-26a that inhibits histone deacetylase 6 (HDAC6) [111].

Therapeutic benefits of engineered exosomes have been reported in stroke. Exosomes have been modified as drug delivery vesicles that penetrate BBB. By promoting the polarization of microglia from M1-type to M2-type, exosomes derived from AMSCs loaded with miR-30d-5p have attenuated ischemic stroke-induced brain injury [112]; exosomes from AMSCs loaded with miR-126 have significantly augmented neurogenesis and angiogenesis, suppressed inflammation, and improved functional recovery [113]. Exosomes derived from BMMSCs overexpressing miR-138-5p have conferred neuroprotection to astrocytes following ischemic stroke via the inhibition of neutrophil gelatinase-associated lipocalin which is involved in brain injury and inflammation [114]. Moreover, BMMSC-derived exosomes have promoted neural plasticity and functional recovery in ischemic stroke via transferring miR-133b [115], making researchers package miR-133b into rat BMMSCs. The exosomes from miR-133b-overexpressed BMMSCs remodeled neurite and strengthened the plasticity of the brain in the ischemic boundary area [116]. Exosomes derived from BMMSCs enriched with miR-17-92 cluster have also increased neural plasticity and functional recovery after stroke, probably through targeting PTEN and modulating the PI3K/AKT/mTOR/GSK-3*β* signaling pathway [117].

Circular RNAs (circRNAs), a novel type of noncoding RNAs, exist in a reverse splicing and covalently closed loop form. CircRNAs could regulate the gene transcription, and hundreds of them are adequately and conservatively expressed in the mammalian brain [118]. The level of CircRNA Scm polycomb group protein homolog 1 (circSCMH1) is significantly downregulated in the plasma of acute ischemic stroke patients. Transfection of GNSTM-RVG-Lamp2b-HA and circSCMH1 plasmids into HEK293T cells produced RVG-circSCMH1-EVs which could target the brain of stroke mice to promote the functional recovery through the delivery of circSCMH1. CircSCMH1 bounded to transcription factor MeCP2 and promoted the transcription of MeCP2's target genes that could modulate neuronal plasticity [119].

Some studies have also loaded small molecular substances and drugs into exosomes to strengthen their therapeutic effects. Yang et al. have demonstrated that exosomes overexpressed with C-C chemokine receptor type 2 (CCR2) significantly alleviated poststroke cognitive impairment via promoting M2 microglia/macrophage polarization [120]. Rosuvastatin as a cholesterol-lowering agent is used to treat BBB damage after stroke [121]. Safakheil et al. have combined rosuvastatin with exosomes derived from BMMSCs, narrowing the infarct volumes and improving neurological recovery [122]. Furthermore, as a natural polyphenol, curcumin could scavenge free radical and control inflammation. Kalani et al. have inserted curcumin into exosomes derived from mouse embryonic stem cells, which restored neurovascular unit following ischemic-reperfusion stroke [84]. Moreover, on the basis of a previous study which has shown that iron oxide nanoparticle−incorporated exosome-mimetic nanovesicles (NV-IONP) could repair SCI [123], Kim et al. verified that in the treatment of stroke, NV-IONP could drastically enhance angiogenesis, suppress inflammation and apoptosis in the site of brain lesion, and improve motor function as well [124]. All the findings above seem to be hopeful for the treatment of stroke.

### 6.2. Therapeutic Potentials of MSC-Derived Exosomes in Neurodegenerative Diseases

Over the last decade, MSC-derived exosomes have been applied for treating neurodegenerative diseases by carrying bioactive molecules to pathological sites (Figure 4).

#### 6.2.1. Therapeutic Potentials of MSC-Derived Exosomes in AD

AD, a chronic and progressive neurodegenerative disorder, is associated with the damaged memory and cognitive function of patients and accounts for more than half of those with dementia. The deposition of A*β* and hyperphosphorylation of tau protein are representative pathological features of AD, leading to inflammation and death of neurons [1, 125]. Animal models of AD can be established by the injection of A*β*1-42 that aggregates into dentate gyrus bilaterally. It has been reported that MSC-derived exosomes could boost neurogenesis and cognitive capacity recovery in a mouse model of AD [126]. Moreover, hucMSC-derived exosomes could shorten A*β* accumulation and alleviate inflammation by modulating the activation of microglia [127]. These exosomes could also inhibit cell apoptosis by carrying miR-223 to modulate the PTEN/PI3K/AKT pathway in an AD cell model [128].

Yang et al. have reported that exosomes isolated from 3D-cultured hucMSCs significantly downregulated the expression of *β*-secretase BACE1 and upregulated the expression of *α*-secretase ADAM10, in order to suppress the production of A*β* in APP/PS1 transgenic mice with the help of their cargoes such as IDE, neprilysin, heat shock protein (HSP) 70, and HSP90 [129]. Additionally, enriched with miR-21, hypoxic preconditioned MSC-derived exosomes have improved the learning and memory capabilities in AD mice [130].

Repeated intravenous infusion is a common route of exosome administration, which causes part of the exosome to be blocked in the liver. To increase the targeting ability of exosomes and avoid nonspecific delivery, dendritic cells were loaded with an expression vector, comprising an exosomal membrane protein Lamp2b and neuron-specific RVG peptide. Matthew et al. transfected BACE1 siRNA into bioengineered exosomes, which specifically delivered BACE1 siRNA to neurons in the brain, significantly decreased the expression of BACE1 and attenuated AD [82]. This study first reported the construction of targeted exosome by transfection expression vector, and it inspired researchers to use exosomes as a wide range of drug delivery tools for treatment. Recently, cui et al. conjugated exosomes derived from BMMSCs with RVG, which markedly targeted to the cortex and hippocampus of the brain, meanwhile decreased A*β* deposition, suppressed inflammation, and improved learning and memory capabilities in transgenic APP/PS1 mice [131]. Exosomes from macrophage pretreated by curcumin could also efficiently deliver drugs to the brain through receptor-mediated endocytosis, preventing the neuronal death and attenuating symptoms of AD by inhibiting phosphorylation of tau protein [132].

#### 6.2.2. Therapeutic Potentials of MSC-Derived Exosomes in PD

PD, the second most prevalent neurodegenerative disorder worldwide, has a series of symptoms including rigidity, bradykinesia, quiescent tremor, and postural instability. PD is correlated with the progressive degeneration of dopaminergic neurons (DAn) and the occurrence of lewy bodies caused by abnormal accumulation of *α*-syn [133].

Dental pulp of human exfoliated deciduous teeth MSC- (DPMSCs-) originated exosomes could restrain the apoptosis of dopaminergic neurons in vitro [134]. Moreover, Kojima et al. have reported an engineered cell that could efficiently produce customized exosomes that delivered therapeutic catalase mRNA into the brain and attenuated neuroinflammation and neurotoxicity in the mice models of PD [135]. Reports on exosomes derived from MSCs in the treatment of PD are relatively scarce; hence, more and deeper researches are required.

## 7. Conclusions and Future Perspectives

Exosomes are emerging as novel players in intercellular communication. The studies of exosomes as potential diagnostic and therapeutic tools for neurological disorders are rapidly growing. Most experimental studies have shown the superior diagnostic and therapeutic effects of exosomes on neurological disorders. However, whether exosomes are the most suitable tools for diagnosis or treatment remains to be tested. The clinical application of exosomes still faces many challenges. First of all, isolation and purification are bottlenecks in clinical application of exosomes. The main reason is that the exosome size and physicochemical properties tend to be confused with other nanoparticles, such as lipoprotein, protein complex, and chylomicron. Ultracentrifugation is the most common method, which is time-consuming and difficult to process large quantities of samples. In addition, there are a large number of high-density lipoproteins (HDL) in plasma or serum, whose density distribution overlaps with that of exosomes, while ultracentrifugation is based on density separation, making it difficult to distinguish HDL from exosomes [22]. Immune-affinity capture can only specifically enrich the exosomes with one or several specific surface proteins, and its yield is low [24]. Ultrafiltration cannot distinguish the chylomicron or lipoproteins from exosomes [25]. Size exclusion chromatography presents the same problem, and the volume of the sample collected is usually large, requiring additional methods to reduce the sample volume [23]. Microfluidic has high requirements on materials and technology and are difficult to handle large samples [27]. It is urgent to explore a uniform and standardized method to obtain exosomes with high purity and high yield. In addition, MSCs exert therapeutic effects under the synergistic action of MSC-derived exosomes and soluble factors [136]. To exclude the contamination of soluble factors, Gao et al. detected cytokines in the supernatant and revealed that there was no soluble cytokines. They found that cytokines were combined with exosomes in an insoluble form [137]. Secondly, in order to increase the therapeutic effect of exosomes and enhance the delivery ability of drugs in vivo, bioengineered exosomes were developed as drug delivery vesicles with the ability to cross BBB, little toxicity, and immunogenicity. However, some drug delivery methods damage the membrane integrity of exosomes. For instance, electroporation, repeated freezing and thawing, ultrasonic treatment, and extrusion made the exosome membrane instantly form small holes to realize load. Hence, the drug loading mode of exosomes remains to be further studied. Last but not the least, our knowledge of exosomes is still limited, and clinical safety issues are not fully guaranteed too. More attention needs to be paid on elucidating the deep mechanisms of exosomes' biogenesis, secretion, and transport, which will provide effective insights to address these issues and ultimately promote the application of exosomes in clinic. Therefore, further exploration of the safety, effectiveness, high yield, high purity, and uniformity of exosomes are required during clinical transformation.

In conclusion, future studies of exosomes will not only further elucidate their roles in the pathogenesis of neurological disorders but also open up new perspectives for clinical diagnosis and treatment of neurological disorders. Exosomes, as a subpopulation of EVs, are believed to become novel tools in the diagnosis and treatment of neurological disorders.

## Figures and Tables

**Table 1:** Comparison of different isolation methods of exosomes.

Isolation technology	Separation principle	Sample size	Advantages	Disadvantages	Ref
Ultracentrifugation	Molecular size, density, and shape	Large	Low risk of pollution, low reagent cost	Expensive equipment, time-consuming, poor biological activity, and integrity of exosomes	[22]
Size exclusion chromatography	Molecular size	Medium	Yield, purity, integrity, and biological activity of exosomes can be ensured	Special equipment	[23]
Immune-affinity capture	Specific binding of antigen and antibody	Small	High purity	High cost, low yield	[24]
Ultrafiltration	Molecular size and shape	Large	Efficient and convenient	Low purity, exosomes may partially remain on the membrane	[25]
Microfluidic	Immune affinity, size, and density	Small	Fast, low cost, convenient, and automated	The selectivity and specificity need to be verified	[27]

**Table 2:** Therapeutic potentials of MSC-derived exosomes in neurological disorders.

Diseases	Sources	Cargos/pathway	Doses	Mode of administration	Species	Results	Ref
SCI	BMMSCs	—	100 *μ*g	Single dose, IV	Rats	Angiogenesis, anti-apoptosis and anti-inflammation	[88]
SCI	BMMSCs	Wnt/*β*-catenin pathway	200 *μ*g	Ten doses (three days apart),	Rats	Antiapoptosis	[89]
SCI	BMMSCs	NF-*κ*B pathway	40 *μ*g	Single dose, IV	Rats	BSCB integrity	[91]
SCI	BMMSCs	NF-*κ*B pathway	200 *μ*g/40 *μ*g	Single dose, IV	Rats	Anti-inflammation	[92, 93]
SCI	hucMSCs	—	20/200 *μ*g	Single dose, IV	Mice	Anti-inflammation	[95]
SCI	BMMSCs	miR-216a-5p	200 *μ*g	Single dose, IV	Mice	Anti-inflammation	[97]
SCI	BMMSCs	miR-133b	100 *μ*g	Single dose, IV	Rats	Neurogenesis	[100]
SCI	BMMSCs	miR-126	100 *μ*g	Seven doses (once daily), IV	Rats	Neurogenesis, angiogenesis and anti-apoptosis	[101].
SCI	BMMSCs	PTEN-siRNA	1.62 × 10^8^ particles	Five doses (once daily), IN	Rats	Neurogenesis and angiogenesis	[102]
TBI	BMMSCs	—	30 *μ*g	Single dose, retro-orbital injection	Mice	Anti-inflammation	[104]
TBI	AMSCs	LncMALAT1	100 *μ*g	Single dose, IV	Rats	Neurogenesis, antiapoptosis and anti-inflammation	[105]
TBI	BMMSCs	miR-216a-5p	100 *μ*g	Single dose, IV	Rats	Neurogenesis and anti-inflammation	[109]
Stroke	UMSCs	miR-26a	1 × 10^11^ particles	Single dose, IV	Rats	Neurogenesis	[111]
Stroke	AMSCs	miR-126	_	Single dose, IV	Rats	Neurogenesis, angiogenesis and anti-inflammation	[113].
Stroke	BMMSCs	miR-138-5p	—	—	Mice	Anti-inflammation	[114]
Stroke	BMMSCs	miR-133b	100 *μ*g	Single dose, intra-arterial injection	Rats	Neurogenesis	[115, 116]
Stroke	BMMSCs	miR-17-92 cluster	100 *μ*g	Single dose, IV	Rats	Neurogenesis	[117]
Stroke	hucMSCs	CCR2	100 *μ*g	Single dose, IV	Rats	Anti-inflammation	[120]
AD	hucMSCs	miR-223	2 *μ*g	—	Cell model	Antiapoptosis	[128]
AD	hucMSCs	Neprilysin, IDE, HSP70, HSP90	12 *μ*g/day	Fourteen consecutive days of Alzet minipump injection	Mice	A*β* peptides clearance and anti-inflammation	[129]
AD	MSCs	miR-21	150 *μ*g	Five doses (two weeks apart), IV	Mice	Anti-inflammation	[130]
AD	BMMSCs	—	5 × 10^11^ particles	Four doses (monthly), IV	Mice	A*β* peptides clearance and anti-inflammation	[131]
PD	DPMSCs	—	—	—	Cell model	Antiapoptosis	[134]

SCI: spinal cord injury; TBI: traumatic brain injury; AD: Alzheimer's disease; PD: Parkinson's disease; BMMSCs: bone marrow mesenchymal stem cells; hucMSCs: human umbilical cord mesenchymal stem cells; AMSCs: adipose mesenhymal stem cells; UMSCs: urine mesenchymal stem cells; DPMSCs: dental pulp mesenchymal stem cells; NF-*κ*B: nuclear factor-*κ*B; PTEN-siRNA: phosphatase and tensin homolog small interfering RNA; LncMALAT1: long noncoding RNA MALAT1; CCR2: C-C chemokine receptor type 2; IDE: insulin-degrading enzyme; HSP: heat shock protein; IV: intravenous injection; IN: intranasal injection.

## Data Availability

The data used to support the findings of this study are included within the article. Readers can open access the data supporting the conclusions of the study.
